# Digital Education for the Deployment of Artificial Intelligence in Health Care

**DOI:** 10.2196/43333

**Published:** 2023-06-22

**Authors:** Fernando Korn Malerbi, Luis Filipe Nakayama, Robyn Gayle Dychiao, Lucas Zago Ribeiro, Cleva Villanueva, Leo Anthony Celi, Caio Vinicius Regatieri

**Affiliations:** 1 Ophthalmology Department Sao Paulo Federal University Sao Paulo Brazil; 2 Laboratory for Computational Physiology Massachusetts Institute of Technology Cambridge, MA United States; 3 University of the Philippines College of Medicine Manila Philippines; 4 Escuela Superior de Medicina Instituto Politecnico Nacional Mexico City Mexico; 5 Department of Biostatistics Harvard TH Chan School of Public Health Boston, MA United States

**Keywords:** artificial intelligence, digital health, health education, machine learning, digital education, digital, education, transformation, neural, network, evaluation, dataset, data, set, clinical

## Abstract

Artificial Intelligence (AI) represents a significant milestone in health care's digital transformation. However, traditional health care education and training often lack digital competencies. To promote safe and effective AI implementation, health care professionals must acquire basic knowledge of machine learning and neural networks, critical evaluation of data sets, integration within clinical workflows, bias control, and human-machine interaction in clinical settings. Additionally, they should understand the legal and ethical aspects of digital health care and the impact of AI adoption. Misconceptions and fears about AI systems could jeopardize its real-life implementation. However, there are multiple barriers to promoting electronic health literacy, including time constraints, overburdened curricula, and the shortage of capacitated professionals. To overcome these challenges, partnerships among developers, professional societies, and academia are essential. Integrating specialists from different backgrounds, including data specialists, lawyers, and social scientists, can significantly contribute to combating digital illiteracy and promoting safe AI implementation in health care.

## Introduction

The health care ecosystem comprises multiple stakeholders, including, but not limited to, health care personnel (HCP), hospital managers, public and private health systems, and end users. With the emergence of artificial intelligence (AI), there is great potential to improve health care outcomes, including reduction of costs and increased access [1]. AI is a transformative technology that can improve medical decision-making, clinical diagnosis, and treatment [2,3]. Image-based diagnosis in radiology, ophthalmology, pathology, and dermatology, genome interpretation, clinical predictions, biomarker discovery, and robot surgery are examples of many AI applications in health care [3-6]. However, traditional education and training of HCP seldom encompass digital competencies [7]. As a result, professionals in the health sector who will be affected by the deployment of AI have minimal exposure to relevant digital education [8] and are currently unable to harness the full potential of implementing AI in health care. In this viewpoint article, we argue that teaching HCP about digital health care is critical for realizing the benefits of AI in health care and for the safe deployment of this technology.

## Electronic Health Literacy

Recent studies have emphasized the importance of teaching digital competencies for HCP, such as their roles in digitalization of health care, knowledge of basic computer science concepts, and legal and ethical aspects [7]. To effectively use AI, HCP will need to understand, interpret, and meaningfully critique the outputs of AI models [9]. This task demands inputs from people with diverse backgrounds such as computer science, mathematics, statistics, law, ethics, social science, and health care. This includes traditional health competencies such as clinical skills and epidemiology [10].

To address these needs, the proposed new medical specialty of “Clinical AI” would expand the more traditional specialty of clinical informatics [11]. Clinical AI specialists would have a leading role in the decentralized approach to safer AI deployment and regulation; they would also continuously review and recalibrate AI models [12]. It has become evident that digital health care teaching is not prevalent in most health-related schools’ curricula [7]; however, incorporating digital health care teaching into the existing curricula presents significant challenges, including an already overburdened curriculum, compartmentalization of the educational program, and time constraints [8]. Knowledge of digital health care is also scarce among fully trained HCP due to cultural unreadiness and a gap between early and late adopters, among other reasons [8].

From our standpoint, electronic health literacy is fundamental for not only the workforce but also other stakeholders in the health care ecosystem, including the end user. Misconceptions and unfounded fears from HCP and patients may jeopardize the real-life implementation of AI systems in health care. Building trust and refuting false beliefs are essential for successful deployment. Moreover, social scientists must analyze the sociocultural implications of software, wearables, and self-care technologies, which are crucial to understanding and avoiding biases and dangerous AI results, ensuring safe implementation [13].

## The Health Care Workforce

HCP must adapt to the changes brought about by the integration of AI in health care. Incorporating digital health competencies in HCP training curricula is undoubtedly challenging, and some approaches have been proposed, including classes, web-based courses, and certifications [8]. Trainees should be taught fundamental AI concepts including taxonomy [9], general aspects of data sets, integration within clinical workflows, concepts on biases, the value of clinical deployment, human-machine interaction in clinical settings, and specific health care applications of AI [8,9,12,14,15]. Werner et al [16] recently reported the successful implementation of a longitudinal, modular course on digital health in medical graduation with positive student feedback. The course included a modularly structured core curriculum and elective courses, beginning with principles of scientific methods, an orientation phase, and concluding with each student selecting a specialization area and preparing a research project as an independent academic achievement [16].

HCP must learn how to collaborate with professionals from diverse backgrounds and how to engage partners outside the system, such as the developers of electronic medical records [9]. They should also learn to access and generate open access data sets for secondary data analysis—such a step being fundamental to the reduction of AI biases and the promotion of fair and generalizable models [17,18].

Since medical training curricula are already saturated, practical training in “Applied AI” has been proposed as a feasible approach to saving time [19]. It has also been proposed that medical information that was once memorized but is now available through AI algorithms should be less emphasized in favor of digital health skills that enable safe and effective interaction with AI technologies [19]. Critical analysis of AI studies, including the ability to identify relevant research questions and recognize the quality of applied data sets, is fundamental [15]. It is also essential to understand the system’s inputs and outputs, metrics, external validation [15], the adequacy of the chosen operating threshold [20], intended use, and epidemiological and socioeconomic considerations. Finally, practical aspects of implementing electronic health literacy into current systems, such as postdeployment studies and recalibration, must be considered [17,21].

Digital competencies could also be taught to graduated HCP through continued education forums on digital health care, scientific meetings, conferences, and datathon and hackathon events. These activities enable collaborative exchanges between HCP and data specialists [22].

## Patients

Patients play a critical role in the successful deployment of health care AI. Despite the reported benefit of AI adoption in multiple fields, patients' misconceptions and false beliefs can lead to mistrust in such systems [5]. For example, a recent survey found that most patients reported being uncomfortable receiving an AI-assisted diagnosis with 90% accuracy, but were incapable of explaining its rationale [23]. Therefore, it is essential to familiarize patients with the benefits and limitations of AI in health care to gain their trust and support.

To promote this goal, a combined effort involving AI developers, HCP, and patient associations could provide a venue for appraising patients on health care AI [1]. Patients not only serve as the end users of health care AI but also constitute partners in the AI enterprise. By generating greater awareness of AI, patients tend to become convinced that by sharing their data, they will improve health care for themselves and other patients [24].

As real-world implementation of AI in health care becomes widespread, greater exposure to AI-driven medical technology will increase patients' awareness and encourage them to consent to sharing their data [24]. Assertive statements from developers reassuring their commitment to data privacy are also essential to develop trust [24-26].

## Conclusions

Electronic health literacy is crucial for harnessing the digital health care revolution. However, the promotion of digital health care is faced with several challenges. For instance, time constraint is a significant obstacle for training HCP on digital health as curricula are already saturated, and adding new content is unsustainable. One possible alternative is to integrate new competencies into existing program components [9]. Furthermore, there is a shortage of professionals with robust data and analytical skills [9], and low incentive to engage busy professionals on this topic. Educating patients on the benefits of AI tools is also challenging. Therefore, stakeholders must be informed about the potential gains, and common myths need to be deconstructed. Partnerships among developers, professional HCP societies, academia, and specialists from different backgrounds, including social scientists, can significantly contribute to advancing the agenda of combating HCP digital illiteracy and preparing the society as a whole to realize the benefits of AI implementation [8].

## Abbreviations

AI: artificial intelligence
HCP: health care personnel
